# Bibliology; or Analytical Reviews of Medical Publications

**Published:** 1819-01-01

**Authors:** 


*An experimental Inquiry into the Laws of the Vital Functions, with some Observations on the Nature and Treatment of Internal Diseases.* By A. P. Wilson Philip, M. D. F. R. S. Second Edition, with Additions. One Vol. Octavo, pp. 384.

"It must be gratifying to an author, (to use the words of Dr. Philip) that a Second Edition of his Work should be called for in less than nine months."

To Dr. Philip, who has been accused of want of feelings of humanity, it must be peculiarly so.

"It is an additional evidence (says he) of the truth of a maxim on which I have ever firmly relied, that a man whose only aim is to act on the principles of correct feeling and common sense, however he may be assailed by the prejudices of some individuals and the injustice of others, will always have the voice of the majority."

We are, indeed, sick of that maukish sensibility that sheds a flood of crocodile tears over the corpse of a rabbit, put to death for the purpose of pathological or physiological research, while it can behold, unmoved, hecatombs of animals every day sacrificed to satiate the appetite of sensuality and gluttony. Let us look around us, and we shall see one half of Nature slaughtering and devouring the other half. Is it not meritorious, instead of censurable, to inflict death on a small animal for the purpose of lessening human sufferings, or prolonging human life?

Dr. Phillip's experiments have been objected to on account of their number; but this arose from the great variety of subjects which his inquiry embraced. Indeed, it appears to be evidently Dr. Philip's aim, on all occasions, to lessen, as much as possible, the sum of suffering on the part of the animal; since a large majority of the experiments was made on the newly dead subject—"a circumstance (adds our author) which my accusers have always overlooked."

Dr. Philip need not have taken any pains to rebut these charges of cruelty preferred by puling philanthropy.—Among all men of good sense and true science they are estimated as they deserve—they are unprofitable declamations written in water, while the deductions resulting from the stigmatized experiments will be perpetuated among the records of our science—Ære perennius.

From causes which need not now be ennumerated, the first edition of this work escaped the analytical department of the Medico-Chirurgical Journal, and the book is too well known and admired to sanction a regular analysis at the present time. But as, "in the eleventh chapter of the second part, the experiments and observations relating to the agency of galvanism, in the former arrangement, were intermixed with several other parts of the Inquiry, and are here presented at one view," we shall select this subject for the present report, convinced that every reader will feel equally interested as ourselves in the deductions and conclusions of our able author.

Dr. Philip conceives, that when the lungs are deprived of a considerable share of their nervous influence, the effect will be a phenomenon or disease precisely analogous to common habitual asthma, in which the breathing is constantly oppressed, more or less, and often continues to get worse, in defiance of remedial aid, till the sufferer is incapacitated for all the active duties of life.

From numerous experiments, he found, that both the oppressed breathing and the collection of phlegm, caused by the division of the eighth pair of nerves, may be prevented by sending a stream of galvanism through the lungs—and this stream may be safely sent through the human lungs, as is proved by repeated trials. These were the circumstances that led Dr. Philip to anticipate relief from galvanism in habitual asthma—a relief which subsequent experience ascertained to result from the measure.

"The time, during which the galvanism was applied before the patient said that his breathing was easy, has varied from five minutes to a quarter of an hour. I speak of its application in as great a degree as the patient could bear without complaint. For this effect, I generally found from eight to sixteen four-inch plates of zinc and copper, the fluid employed being one part of muriatic acid, and twenty of water, sufficient. Some require more than sixteen plates, and a few cannot bear so many as eight; for the sensibility of different individuals to galvanism is very different. It is curious, and not easily accounted for, that a considerable power, that perhaps of twenty-five or thirty plates, is often necessary on first applying the galvanism, in order to excite any sensation; yet, after the sensation is once excited, the patient shall not perhaps, particularly at first, be able to bear more than six or eight plates. The stronger the sensation excited, the more speedy in general the relief. I have known the breathing instantly relieved by a very strong power." P. 334.

"The galvanism was applied in the following manner. Two thin plates of metal, about two or three inches in diameter, dipped in water, were applied, one to the nape of the neck, the other to the pit of the stomach, or rather lower. The wires from the different ends of the trough* were brought into contact with these plates, and, as observed above, as great a galvanic power maintained, as the patient could bear without complaint. It is proper constantly to move the wires upon the metal plates, particularly the negative wire, otherwise the cuticle is injured in the places on which they rest." P. 335.

The process was discontinued as soon as the patient admitted that his breathing was easy. In several very chronic cases, the patient was as quickly relieved, as in more recent ones, proving that no permanent or organic lesion had affected the pulmonary structure. In regard to the regular spasmodic *periodical* asthma, returning in violent paroxysms, with intervals of free breathing, Dr. Philip does not expect, and has not found the galvanism of any material service.

Of the cases of habitual asthma, many occurred in work-people of the city of Worcester, who had been obliged to abandon their employments in consequence of the complaint; and some of them, from its long continuance, had abandoned all hopes of returning to regular work. "By the use of galvanism they were relieved in different degrees, but all sufficiently to be restored to their employments." The Doctor saw many of his patients some months after leaving off the galvanism, who, nevertheless, continued to work without any inconvenience.

Dr. Philip is inclined to think, that in inflammatory complaints, it would be injurious; and also in dyspnœa arising from dropsy. Habitual asthma is also frequently attended with a torpid liver, and some fullness and tenderness on pressure near the pit of the stomach. If the last be considerable, it ought to be relieved previously to the use of the galvanism. This last, he thinks, is beneficial in affections of the digestive organs, unaccompanied by any inflammatory action.

"I have repeatedly seen from it the same effect on the biliary system, which arises from calomel; a copious discharge from the bowels coming on within a few hours after its employment. This seldom happens, except where there appears to have been a failure in the secreting power of the liver, or a defective action in the gall-tubes." P. 339.

The cough which so commonly attends habitual asthma, did not counter-indicate the use of galvanism—the latter, indeed, lessens the accumulation of phlegm in the lungs.

* Our author found a trough of the old construction more effectual in restoring the due action of the lungs than the improved pile

In some very chronic forms of phthisis, where the symptoms had lasted several years, and habitual asthma had supervened, the relief obtained from galvanism was very considerable; nevertheless, in ordinary cases of phthisis, this would be improper. Where there is considerable tendency to inflammation in the system of an habitual asthmatic, this should be removed by evacuants, anterior to the use of galvanism. This measure was seldom used more than once a day.

"About a sixth part of those who have used it, appear, as far as we yet know, to have obtained a radical cure. It in no case failed to give more or less relief—it failed to give considerable relief only in about one tenth." P. 343.

Dr. Philip cannot speak to the effects of galvanism in dyspepsia, as he has not yet made sufficent trial of the remedy. Our author relates the particulars of a few of the most, and of the least successful cases of habitual asthma in which the remedy was tried. Some of these we shall condense.

*Case I.* R. Morgan, ætat. 50, a blacksmith, had laboured under severe habitual asthma for seven months, with various degrees of intensity, but without any clear interval. He was also much troubled with cough and expectoration. The first application of the galvanism relieved him.

"He was galvanized only for three days, about ten minutes each day, before he declared himself to be perfectly well."

After its third application he performed as hard work, and with as much ease, as he had ever done. He remained free from the disease for several weeks, till it was renewed by a fit of intoxication. Galvanism relieved him as readily and effectually as at first. It is now ten months since he first used this remedy, during which he had several returns of dyspnœa, but it has never been so severe as before he was galvanized.

*Case II.* Mary M'Konchy, ætat. 28, a gloveress, had been affected with habitual asthma for four years, without deriving any material advantage from medicine. She had some languor in the biliary system, but little inflammatory tendency. The breathing was, in a few minutes, rendered easy by galvanism, and after the second application of it, it remained so. She now experienced no inconvenience from exercise, which had not at any time been the case for four years.

"In about three weeks after she had been galvanized she experienced some return of the dyspnœa. It was wholly removed by a blister, which had been often tried, previous to her being galvanized, with but little and very temporary relief. She complained of a sense of sinking at the stomach for some time after the use of the galvanism, which was removed by carbonate of iron and bitters. P. 350.

*Case III.* Isaac Radley, ætat. 68, a labourer, and formerly a soldier, dates his asthma from sleeping in camp in Holland fourteen years or more ago. He has never been able to walk at the usual pace since, without bringing on dyspnœa. He has lately been forced to abandon work altogether. He had a most severe fit which lasted half a year. He was relieved in a few minutes by the application of the galvanism, and he could perceive its beneficial effects for twenty-four hours after its application. It was used daily with the same immediate relief. Its permanent good effects gradually increased, and after he had been galvanized about ten minutes each day, for between two and three weeks, his breathing remained quite easy. He could now walk or even run; his digestion improved, and he has now remained two years without any return of the disease.

On what principle shall we explain the *permanency* of relief afforded in these cases? Dr. Philip conceives, that there are two ways in which an organ may be deprived of its nervous influence; either by a failure of due action in the brain and spinal marrow, the sources of nervous influence, or a failure of due action in the nerves of the organ affected by which this influence is conveyed.

"Now we have reason to believe, that habitual asthma arises not so much from a fault in the brain and spinal marrow, as in the nerves of the lungs; because did the degree of dyspnœa, which we often witnessed in this disease, arise from failure in the general source of nervous influence, this failure must be sufficient to appear in the derangement of all the nervous functions; whereas in habitual asthma, we often find the functions of the lungs alone affected; and when general failure of the nervous influence is observed, it is evidently the effect of impeded respiration, appearing only after the latter has continued for some time, and varying as it varies. The effect produced by galvanism, when it performs a cure in habitual asthma, therefore, does not appear to be its having occasioned a permanent supply of nervous influence, but its having cleared, if I may use the expression, the passage of this influence to the lungs. It is not difficult to conceive, that such an obstruction may exist in the nerves as cannot be overcome by the usual supply of nervous influence, though it may yield to a greatly increased supply of it; and that it may, in some cases, continually recur in an equal or diminished degree, while in others, being once removed, the tendency to it may cease. What is here said is well illustrated by the effects of galvanism in apoplexy. We know, that in this disease, the dyspnœa arises from a failure in the source of nervous influence, and the relief obtained from galvanism corresponds with the views afforded by the experiments which have been laid before the reader. While the galvanism passed through the lungs the dyspnœa was as much relieved as in habitual asthma, but when it ceased to pass through them, the relief lasted no longer than was necessary for the re-accumulation of the phlegm." 357.

Into the mysterious transaction recorded at the end of the work, we shall not enter. We have no doubt of the triumph of *truth;* and that truth and accuracy are on the side of Dr. Philip, we have as little doubt. It would be an insult to the understandings of our readers to say any thing of the merits of a work, on which the public approbation is stampt in such indelible characters. We can only add our humble suffrage to the general voice, and unite in hoping, that the man who has so ably contributed to enlarge the boundaries of physiological and pathological knowledge, will persevere in his praise-worthy investigations, and thereby insure the gratitude and veneration, not only of the present, but of future ages.

P. S. Since the above pages were written, we have learnt, that in some public institutions of this metropolis, Dr. Philip's plan of treating Asthma by Galvanism has been tried, and with the greatest advantage. We therefore hope, that it will be extensively adopted throughout the kingdom.

---

### VII.

*Practical Observations on the Nature and Treatment of Marasmus, and of those Disorders allied to it, which may be strictly denominated Bilious.* By Joseph Ayre, M. D. &c. One Vol. Octavo, pp. 250. London, 1818.

Men of talents, like men of wit, are not only ingenious in themselves, but are often the cause of genius in others. Dr. Ayre and Dr. Armstrong were associates in their early studies: they are now candidates for public favour. But emulation has not trenched upon the bounds of friendship.

Dr. Ayre dedicates the present work to his quondam fellow student, whose bright example has probably stimulated him to follow in the path where his friend has so ably led the way. We even fancy we can trace a kind of half-studied imitation of the "Practical Observations on Typhus," in the present volume, at least as to manner and style; and although the copy falls very far short of the original, it is yet a production of much merit, and evinces no trifling talent for observation and discrimination. We shall, therefore, present a copious analysis of the work to our readers, with such observations of our own, as experience may suggest.

Dr. Ayre complains, and perhaps with justice, that the hepatic system is referred to, almost indefinitely, as the origin of every disorder, by many practitioners, and *that* too where the stomach merely is concerned. He thinks also, that they have regarded these affections of the hepatic system too much in the light of inflammatory or *organic* diseases. His object, therefore, in the work before us, is to point out the nature of these and similar errors in diagnosis, together with the practical consequences resulting from them. In the view which our author takes of *marasmus infantum*, he has identified it with the "bilious disorder" of adults, or "with that which Mr. Abernethy has termed a disorder in the *digestive organs*." He considers it as

"An impeded biliary secretion, which gives rise to a venous congestion of the liver, and as standing, in consequence of this congestion, in the relation of a cause to the cholera morbus, and to the idiopathic hæmatemesis and melena of nosologists." viii.

Our author's views then of marasmus led him at once to the mode of practice, "which consists in removing the congestive state of the liver, by renewing the healthy and natural functions of that organ." ix.

Marasmus, known by the names, "febris hectica infantum," "infantile remittent fever," "worm fever," &c. has been generally regarded as peculiar to children, but our author believes, that

"There is no essential difference between the marasmus of children, and that disorder of adults, which may be strictly denominated bilious, either in the nature, or in the causes, or in the means of cure." P. 2.

Bilious disorder and marasmus are therefore adopted as synonimous and convertible terms, and three separate views of it are presented, as it appears in infancy, childhood, and adult age. Our author divides the disease generally, into the acute and chronic forms, the *former* attended by a considerable loss, or by an absolute extinction of the appetite, with thirst and fever, the *latter* being distinguished by a morbidly craving appetite, unattended by either thirst or fever.

We shall endeavour to condense Dr. Ayre's symptomatology of the disease; and the more so, because we think him in many places needlessly prolix and minute, a feature that not unfrequently obscures his doctrines at least, if not his therapeutics.

In infancy, this disease produces languor; sleepiness by day and restlessness by night; morbid appetency for food, which, as the complaint advances, veers round to loathing of food. Towards evening, increased frequency and labour of breathing, with some stupor, starting, and heat of the body simultaneously, with coldness of the feet and hands. If not relieved, a low degree of convulsion, called by nurses "inward fits," supervenes, terminating at length in the strong and more fatal convulsion.

If the infant be a few months old, or of a vigorous constitution, its bowels will sometimes become spontaneously loose, immediately after the commencement of the complaint, and then its craving appetite may continue, with occasional interruptions, for many weeks, without any considerable aggravation of the complaint, the alvine discharges being always depraved; many-coloured, slimy, yeasty, or offensive. The flesh gets flabby and wastes; tongue white, sometimes aphthous; occasional spasmodic cough in the evening, with what is termed "stuffing." Eruptions sometimes break out about the nose, mouth, or ears, or a rash on the body, with a temporary relief of the more urgent symptoms.

After a time the looseness becomes less serviceable, and the matters more and more unnatural in colour, with straining. The craving appetite changes to the opposite state, with a considerable increase of the fever, attended by restlessness, with intervals of stupor terminating in convulsions. Temporary alleviations of the fever are occasionally observed from a return of the looseness; but the wasting of the flesh and strength proceed, the infant dying at length in a state of extreme emaciation and weakness.

When the age is advanced beyond the fourth or fifth year, there is some variation observable, the chronic form of it running less readily into the acute, from the diminished tendency to febrile and convulsive action in the system. In these subjects then, the chronic form approaches insidiously, at first merely as a craving for food. In time the countenance loses its animation, and the muscles their natural energy of action. He complains of being chilly and tired, and of having an aching pain in the knees and lower parts of the thighs. He is dull and fretful; breath fœtid; itching of the nose, with discharge; slight pain or dizziness in the head. Towards evening desire to go to bed, with heavy sleep in the night; tongue white; bowels somewhat deranged. As the disorder advances, there is a disposition to faint, with nocturnal perspirations about the head and neck. Moaning, starting, and grinding his teeth in sleep, tickling spasmodic cough in the evenings, with occasional retchings; hurried and quick breathing during sleep; bowels alternately loose and costive, the craving appetite failing as the looseness abates; skin harsh and dry; wasting of flesh and strength; occasional vomiting of his food.

Should any of the remote causes, as cold, improper diet, &c. be now applied, the acute form suddenly supervenes. Nausea, thirst, quick pulse, and high fever in the evening, with slight remission in the morning. The child raves or screams in his sleep, awaking in much distress; there is often considerable pain in the head, stomach, and bowels; tongue foul; urine scanty, high-coloured, and turbid; bowels constipated; and when moved by medicine, yielding feculent matter of a dark, slimy, yeasty appearance, and of a sour and highly offensive smell. The complexion is sallow, and the whole countenance appears languid, sunken, and somewhat fatuous.

*In the Adult.*—Here the chronic form generally creeps on in the same gradual manner, and with nearly the same symptoms, as in the child. The craving appetite is often the first noticed symptom, the patient complaining that the food does him no good; that he has an empty sinking feeling at the stomach, which is only temporarily relieved by food. He has listlessness and drowsiness, with chilliness, aching in knees and ancles; slight vertigo; dimness of sight; inaptitude for mental exertion; dejection of spirits from inadequate causes; great fatigueability from moderate exercise; unrefreshing sleep. In progress, the sleep is much disturbed; the complexion becomes decidedly sallow, particularly about the forehead, and back of the hands; the eyes lose animation. Loss of strength succeeds, generally with wasting of flesh. Constipation of the bowels; the fæces being always unnatural; frequently of a black green and slimy appearance, with offensive odour. The urine is commonly turbid, high coloured, with lateritious sediment; tongue dry and furred in the morning, becoming sometimes nearly clean after breakfast. The pulse undergoes little alteration, excepting in irritable habits, where it is quickened. There is seldom much thirst; but there is generally a little heat about the head and breast, in the early part of the night, with a ready disposition to perspire profusely, at the same time that the feet are cold. In the fore part of the day the patient thinks himself well, but falls off towards mid-day, and becomes more indisposed as the evening approaches. There is seldom cough in this complaint as it affects adult age.

This chronic stage will last for months or even years, with only an occasional abatement or aggravation of the symptoms, when at length it will pass suddenly into the *acute* state. When this last is fully formed, the very sight and smell of meat have a sickening effect. There is either an oppressive feeling, or an acute pain in the region of the stomach, or in one of the sides; generally the left; or in the bowels striking to the back; excessively restless nights; some heat of skin; with thirst towards evening and during night; furred tongue; scanty, turbid, and high coloured urine; fecal discharges like yeast, or dark, and even perfectly black, resembling tar in colour, and of exceedingly offensive smell.

It is not to be understood, that all these symptoms are present in every case, though a very considerable proportion of them will be found so. Our author thinks, that this disease is often liable to be confounded with difficult dentition, tabes mesenterica, hydrocephalus internus, dyspepsia, hysteria, hypochondriasis, chlorosis, &c. and truly we believe, that many of the above are the same with the disease under consideration. But the more important diseases, Dr. A. conceives, with which marasmus is likely to be confounded, are, the anasarca of debility, phthisis pulmonalis, and the organic and inflammatory affections of the liver. The discriminating symptoms we cannot stop to notice, but refer our readers to the work itself. We may, in passing, however, add our suffrage to that of our author, in stating that many, very many *functional* diseases of the liver are erroneously regarded by medical men as *organic* changes.

" Such views, when thus reduced to practice, cannot fail to exhibit their fallacy, since they are founded on the error of regarding the liver as diseased, when it is only impeded, or disordered, in its action; and of employing means for reducing the force of the arterial action of the organ and of the system, instead of those proper for renewing and sustaining its secretory function. To those of my readers, who may have imbibed such views, I would take the liberty to recommend the instructive practice of dissection; for from it they will soon learn for themselves, to distrust such speculations. In prosecution of their examinations, they will find but very few cases of diseased structure of the liver, compared with those which they had perhaps classed as such; for, however the contrary opinion may be generally entertained and expressed, a disease of the liver, according to the observations which I have made from such repeated examinations, is comparatively rare." 39.

We do not go quite this length; but we had long ago concluded that the diseases of function were infinitely more common, and perhaps more important, than organic diseases of the liver.

"That tendency, however, in the liver, to assume organic disease, which may be considered as comparatively rare in the chronic form of the bilious disorder, may be justly regarded as constituting a prominent feature in the severer forms of the acute one. The liver, in these cases, frequently acquires an increase in its bulk, so as to be readily felt externally." P. 45.

The patients in whom Dr. Ayre met this disease always had it as an effect of the acute state of the bilious disorder, the symptoms in the latter being severe. Here the fæcal discharge is of a yeasty colour; the urine excessively high coloured, turbid, and scanty—and the pain in the region of the stomach peculiarly severe during the night, and commonly increased by the recumbent posture. Bleeding and blistering produced no good effects in these cases. Dr. Ayre considers this enlargement of the biliary organ as not depending on the mere congestion of its vessels, but probably on the deposition of a serous or coagulable fluid into the parenchymatous substance of that viscus. In an early stage, and before the new matter acquires an organic structure, the removal of the disease is not difficult, as this affection differs materially from those tuberculous disorganizations which have been held forth to public view as the most common, when, in fact, they are the most rare forms of organic diseases of the liver.

Dr. Ayre states, that in the epidemic fever which prevailed at Hull in 1817, 18, full one fourth of the patients were affected with cough from bilious irritation. From the tightness and oppression about the chest, it exhibited many of the characters of pneumonic inflammation.

"The function of the liver was so remarkably affected from the very commencement, and during the whole course of the fever, as to give to the disorder a complete bilious character. The stools were either black or clay-coloured; the cough and the other symptoms of the fever subsiding only as they recovered their natural state."

Diarrhœa never proved salutary unless of a bilious character. The cough and fever were aggravated by opiates, and reduced only by free purgatives, which promoted the secretion and the descent of the bile. The fever was decidedly contagious; and we may remark, en passant, that our author attributes the comparatively small mortality of this epidemic among the poor to their destitute condition, for no instance of fever ever came under his care, where an abstemious regimen was more necessary than in this. Even during the convalescent state, nearly the same rigid prohibition of food and stimulant drink was needful; for where it was neglected, relapses were common.

Dr. A. begs to observe here, that his omission of venæsection in this fever is not to be considered as conveying a tacit disapprobation of the use of the lancet in fevers in general.

"On the contrary, (says he) I esteem the general conviction, now prevailing, of its utility in several forms of continued fever, when employed early, as one of the late triumphs, atchieved by science and observation, over prejudices long established." P. 54.

Dr. Ayre's principal dependence, in this epidemic, was placed on those medicines "which procured biliary evacuations from the bowels, by promoting the secretion and descent of the bile;" by mild diaphoretics, by a cool regimen, and by a spare diet.

But to return. Dr. Ayre does not disdain to take the morbid sympathies into consideration.

"A considerable number of the symptoms of the bilious affection, are produced by the operation of that law of the animal economy which we term sympathy; for, besides the general and local disturbance which is observed to arise directly from the disordered actions of the liver, and the other chylopoietic viscera, there are several important affections produced through the sympathetic connexion subsisting between these organs and different parts of the system, whereby an irritation, present in the former, is communicated to parts of the body with which they have no local nor apparent relation." P. 57.

Among the various sympathetic affections of this kind, Dr. Ayre enumerates those serous effusions which occasionally take place in the cellular membrane of the extremities, or even in the cavities of the chest or abdomen.

"When this irritation is directed to the membrane lining the larynx and trachea, it gives rise to the bilious cough; which, in many cases, very strongly resembles some of the forms of phthisis pulmonalis."

The resemblance, indeed, between the two diseases, is very strong; and has deceived some of the most observant practitioners. The breathing, however, in the sympathetic cough, though hurried in the evening, from the accumulation of phlegm, or on the accession of fever, is generally calm and natural in the morning; and although the pulse is not invariably rapid in phthisis, excepting in that from tubercles, yet it is nearly constantly so; and has always a preternatural degree of strength and wiriness in the evening, and even during the day, which is seldom met with in the same degree, in the simulated phthisis under consideration. The discharges from the bowels, in the real pulmonary consumption, are commonly healthy, whilst in bilious disorders they are uniformly of an unnatural colour and fœtor. The hope and animation too, attending the former, are wanting in the latter.

But it must be borne in mind that, where a mucous membrane has been long under the influence of a sympathetic irritation, it may, and does take on purulent secretion, ending in ulceration.

Among the morbid sympathies of bilious irritation may be remarked those of the head. Pain and dizziness, and a cloudiness of vision, with dark spots passing before the eye, attend the chronic form of the complaint, together with a decided failure in the powers of the mind. This irritation, Dr. A. believes, is often propagated to the brain in children, and there produces symptoms exactly resembling hydrocephalus internus. This we believe, and more too; for we are of opinion, that these symptoms are not merely resemblances, but realities. This, indeed, in a subsequent page, Dr. A. virtually admits.

"There is, in fact, often considerable difficulty, especially in infancy, to determine where the symptoms proper to the marasmus terminate, and those belonging to hydrocephalus internus begin." P. 78.

This is acknowledging that they are but two links of the same chain; or, as he in the next page observes,

"When the irritation, (cerebral) in fact, has reached no higher state than what is met with in marasmus, it is removable; but when it exceeds this, and rises to that point in which a fluid is poured out into the ventricles, it becomes, I believe, irremediable."

Notwithstanding the uncertainty which still obtains respecting the actual effusion in hydrocephalus, our author ventures to lay down certain pathognomonic marks of the disease.

"Where there are sudden screamings, and the carrying the hand to the head, with an importunate desire to have the head kept low, and pressed between the occiput and forehead, and a delirious raving in the day time disproportioned to the fever, with the double vision, or blindness, and the slow intermitting pulse, the practitioner may assure himself, with little risk of error, of the immediate approach, or the presence of that fatal disease." P. 8.

We lately attended a child who had not, till the last moment, a single symptom of those above enumerated. A very bad state of the bowels, a sickness at stomach, and an evening exacerbation of fever, were all the symptoms we could observe, or the mother describe. The eye was perfectly natural, and we never could discover an intermission or irregularity of the pulse. Yet, on dissection, (and we demonstrated the brain to two medical gentlemen) there was great effusion in the ventricles, and at the base of the brain, with inflammation and congestion of its vessels. What are we to say to this? Indeed, we have opened so many heads where the brains and their coverings were inflamed, or effused with serum, and yet where the characters of the disease were so faint during life, that we almost despair of pathognomonic symptoms in cerebral affections.

But it is not, Dr. A. thinks, to the secreting and exhaling surfaces only, that this sympathetic irritation is confined.

"The cellular membrane is often the seat of it; and indeed it is probable, that many of those abscesses which take place in deep-seated parts, and even those which are discovered in the brain, derive their origin from the same source; for there is scarcely any part of the body that seems exempt from the influence of biliary irritation, nor scarcely any of the morbid actions, local or general, of the system, which it does not sometimes produce."

Dr. A. knew a case of mania from this cause, which continued unabated for three weeks, and which was immediately removed by copious tar-like evacuations from the bowels. We have seen three instances of this kind. The various and changing forms of scrofula originate, Dr. A. thinks, from this source; and also those herpetic eruptions about the ears of children. They are all relieved by correcting the disorder of the digestive functions, and this principally by destroying an irritation which sympathetically excites the local disease.

### *Pathological Observations.*

The functions concerned in digestion, chylification, biliary secretion, &c. are all so catenated, that when one becomes deranged, the circumstance is liable to throw one or more of the others into disorder. Thus the stomach being the primary seat of derangement, an imperfect or morbid stimulus may be given to the liver, and *vice versa,* and thus a series of irregular actions in the chylopoietic viscera may ensue. Whether these deranged states of the stomach, when primary, are of the nature of dyspepsia, Dr. A. does not pretend to know; nor can he tell whether the stomach be always primarily affected, or only occasionally. It seems to him probable, however, from the tough phlegm, which is early and constantly present in the stomach and bowels of those affected with marasmus, that it has a considerable influence in many cases, in aggravating, if not in causing the complaint. This he thinks may be inferred from the beneficial effects that are generally found to result from an emetic exhibited at an early stage of the complaint, when much of this phlegm is always thrown off the stomach.

"But upon whatever hypothesis we may found our explanation of the *origin* of marasmus, it appears to me indubitable that its seat is always in the liver, and that the means directed for the relief of the deranged actions of this organ, are adequate to restore the other disordered functions to a healthy state." P. 96.

Our author justly conceives, that the merely loaded and constipated state of the bowels is not sufficient to account for marasmus, as it appears in children. The acute form of the complaint sometimes comes on suddenly from cold or other causes, and purging is generally inadequate to its cure—the relief, indeed, is often afforded many hours before an evacuation takes place from the bowels. Neither is it till the fæces have acquired their natural bilious colour, that the disorder itself is relieved. Dr. A. argues thus:—The bile is the fluid which gives the tinge, in health, to the fecal discharge; the other gastric and intestinal secretions being nearly colourless.

"Any change, therefore, form the natural appearance, must result from a change in the bile."

This is, generally speaking, correct; but although attaching every degree of importance to the biliary secretion, we are convinced from close observation, both in ourselves and others, that the morbid secretions from the intestinal canal often simulate diseased secretions from the

This deterioration he justly supposes must take place either during the secretion, or after its passage into the bowels. To the *former* mode this change, of course, is mainly attributable—an inference deducible from the well known fact that in *organic* diseases of the liver, we have, in general, similar privations of its peculiar secretion. Even the green colour of the bile is not always, perhaps indeed, not generally, the consequence of a commixture with acids, or calomel in the primæ viæ, since dissection often shows us a similar fluid in the gall-bladder; and in the bilious vomitings which so frequently occur in sultry regions, we find the hepatic secretion exceedingly disordered without any stay in the stomach or bowels, and often under circumstances where no contact with acids or medicines could be suspected.

" In those cases where the stools are clay-coloured, there is incontestible evidence afforded of an interruption in the secretory process; for, generally, neither the skin, nor the secretion by the kidnies exhibit [exhibits] any greater signs of the absorption of bile into the system, than are manifested under other states of the alvine discharge." P. 100.

To this passage we cannot give our entire assent. We have generally observed, in deficient secretion of bile, some sediment in, or turbid state of, the urine. Dr. Ayre instances jaundice, to prove that where bile does not come down into the bowels, the stools are never black. To the question, therefore, what is the nature, and what the source of the exceedingly black stools so frequently met with in this disorder? our author answers, " from every observation that I have been enabled to make upon the appearances of the fæcal discharges, and from every consideration I have been able to give to the subject, I feel warranted in concluding, that they derive their black colour from an admixture of blood with them;" " and that this blood is derived from the extremities of the branches of the vena portarum." The two states of the disease already described, acute and chronic, Dr. A. thinks dependent upon different conditions of the liver—" there being a congestive state of the vena portarum and its branches in both forms of the complaint; but in the acute one, there is a higher degree of this congestive state, giving rise, in its turn, to a certain degree of venous congestion in those organs of the abdomen whose circulation is associated with that of the liver." A torpor in the secretory vessels of the liver, Dr. A. observes, will occasion an accumulation of blood in the portal circle, a position which has been brought forward of late by others, anterior to Dr. Ayre's publication. In elucidation, our author instances the female breast; which, when gorged with blood, will terminate in suppuration, if not relieved by artificial means, as the local abstraction of blood, or naturally, by the renewal of the secretion.

"But [here] purulent secretion is the result of *arterial* action, and *cannot* take place in vessels whose character and structure are *venous;* and which, as far as our observations go, are *incapable* of taking on the proper inflammatory action." P. 104.

We read this passage over several times before we could believe the evidence of our senses. Are we to be told that the venous structure of the liver, which constitutes nine-tenths of its bulk, is incapable of inflammation and suppuration, after seeing the whole substance repeatedly broken down into one mass of suppuration, and that in the course of a few days' illness? There is perhaps no viscus in the body more prone to suppuration than the liver; and as it is almost entirely nurtured, or at least supplied by venous blood, and composed of venous tubes, it is most astonishing that Dr. Ayre should be capable of bringing forward so untenable a doctrine as the above.

We agree with him, however, that the mode by which venous congestion of the liver can be permanently removed, is by a renewal of the biliary secretion.

"Where this does not take place, and the congestion is considerable, there may be relief afforded to the organ by the escape from it, through the Pori biliarii of a portion of blood, which, by passing along the ductus communis, will descend into the duodenum." P. 104.

The doctrine of an increased secretion of bile being the *natural cure* instead of the *cause* of cholera morbus, though impugned at first as a "damnable heresy," is now beginning to appear a rational opinion.

"Of the first of these forms, we have a striking instance afforded in the cholera morbus, which, as consisting in a copious secretion of bile, *is produced by those efforts which Nature makes, and by which she often succeeds, to free herself from disorder.* The secretion, however, is in excess, and produces, more or less of disorder; *for the first effect of the morbid cause having been to check the secretory function, the reaction which ensues occasions it to become excessive.*" P. 105.

But in some cases, as was before observed, our author thinks that the congestive state of the liver, instead of being relieved by increased secretion, obtains a temporary relief by the escape of blood from the branches of the veua portarum.

"This effect I have seen in a variety of instances in this disorder, since my attention became more particularly directed to inquire concerning the cause of fæcal discolouration, tracing the changes in the colour of the fæces from a deep brown to black, and from this last to the complete venous hæmorrhage; this state yielding, as the cause was removed, to the black, and this to the lighter shades, until the healthy colour was resumed." P. 107.

When the venous blood thus discharged passes through the bowels, it constitutes, he thinks, the melæna; which, if it meet with obstruction in its descent, may regurgitate into the stomach, and there produce hæmatemesis. Dr. A. thinks that this discharge has been erroneously considered to arise from rupture of the vessels of the stomach; when, in reality, it is to be viewed and treated as resulting from a congestive state of the liver. On these principles, he conceives, that Dr. Hamilton's *practice* is good, though his *doctrine*, in respect to this complaint, is erroneous. Indeed, dissection after death, from hæmatemesis, has seldom revealed any rupture or erosion of the gastric or intestinal vessels, a striking example of which may be found in Heberden's Commentaries, p. 403. From these and other considerations, our author infers, that melena, as well as hæmatemesis, are only modifications of the cholera morbus, arising, like this, from a congestive state of the secretory vessels of the liver.

In recapitulating these doctrines, we find the following as the sixth position; on which we mean to make some animadversions.

"That whilst this congestive state of the liver produces an assemblage of symptoms, resembling, in many points, the acute inflammation of that organ, it differs essentially from that state in many important particulars. For in the acute inflammation of the liver, it is the arterial action of the organ that is excited, and the congestion (if the expression be allowable) is arterial; the secretory function of the organ, from its being carried on by a distinct class of vessels, partaking only secondarily, and partially in its effects; whilst in the venous congestion of the liver, consequent upon an interruption in its secretory action, the arterial system of the liver is necessarily but little, if at all affected; the congestive state in that organ being, in all probability, limited to the vena portarum and its branches." P. 123.

In the first place, how does Dr. Ayre prove that the process of secretion is limited solely to the venous system of that organ? Most of our best physiologists think quite the contrary—for instance, Bichat; which shows, at least, that it is only conjecture on either side. But secondly, as the vena portæ takes on the office of an artery, (which no other vein does) who can prove, or even offer a rational reason, why it should not be capable of taking on inflammatory action? In the third place, we should be glad to know if the *venous* capillaries of an inflamed part are unconcerned? Lastly, as the blood from the artery and vein in the liver *meets*, or rather *mingles* in the secretory structure of the organ, and is returned by the *same channels* (the venæ cavæ hepaticæ) to the heart, we would ask, how an interruption in secretion can congest exclusively the venous system, leaving the arterial current free to pass on? In short, we think this the most hypothetical and faulty piece of pathology in the work—neither founded on facts nor supported by ingenuity.

*Remote Causes.* These are, cold; irregularities in diet; excess in the use of spirits; the impure air of crowded or close situations; certain eruptive fevers; sedentary employments, &c.; on each of which, Dr. Ayre makes many sensible and highly interesting remarks.

*Treatment.* However the vis medicatrix naturæ may be ridiculed by modern physicians, it is nevertheless incumbent on us to watch those efforts which Nature makes for her relief; for we may gather important information from these spontaneous movements, both in regard to the pathology and therapeia of diseases. In the disease under consideration, Dr. A. has endeavoured to show that an interrupted secretion and congested vascular system of the liver formed the prominent features; and that where a renewal of the secretion does not take place, a temporary, or even sometimes a permanent relief is afforded to the organ and to the constitution at large, by a hæmorrhage from the congested vessels, in the form of hæmatemesis, melena, hæmorrhoidal flux, &c. To imitate these sanative efforts of Nature, therefore, as far as they are imitable, and to supply what they fail to afford must be, thinks our author, and think we, the paramount object of the practitioner.

His three general indications stand thus:—1st. To correct the disordered action of the liver, and remove the congestive state of that organ; 2d. to cleanse the bowels of their morbid secretions; 3d. to lessen or avoid the causes.

To fulfil the two first indications, those purgatives must be selected which have "an immediate and specific action on the liver, and are chiefly purgative by promoting the secretion and descent of the bile." Of these, "the mild preparations of mercury are the chief, and calomel may be considered as the one, whose effects will be found most uniform and most to be relied on." To this we subscribe, so far as children are concerned, or adults in the *acute* stage of the disease; but in the reverse of these circumstances, we have found the quicksilver pill, upon the whole, the most generally useful, and the least productive of debilitating effects on the constitution.

"When a patient, who labours under a derangement of the biliary function, takes a small dose of calomel at bed-time, it commonly happens that, instead of the restlessness of former nights, he sleeps more calmly and soundly than usual, and awakes in the morning with a conviction that he owes his rest to an opiate. This fact I so frequently observe, as to be fully satisfied that the restlessness in this complaint depends upon the presence of some disordered actions, which the medicine relieves. And that these actions are those of the liver, and that the final change produced is in the functions of that organ, appear from the alteration which is observed in the motions; for previously to the use of the medicine, they are perhaps black, or of the colour of yeast, and of an unnatural fœtor; whilst during its use, they gradually acquire their proper and healthy appearance." P. 161.

Dr. Ayre justly observes, that the power of this medicine over the secretory functions of the liver is not confined to the augmentation of its activity when torpid; since "it is equally efficient to reduce the secretory action when in excess, its tendency, when acting, being to restore the actions of the liver, whether deficient or excessive, to their natural and healthy state." Our author recommends the doses to be very small, so as not to act in a purgative character.

"In ordinary cases of the disorder, a dose once in twenty-four hours is sufficient; but where the symptoms are urgent, it is often necessary to give it more frequently, abstaining from the use of purgatives until after the last dose of the calomel has been taken." P. 193.

When the bowels are constipated, and there has been much irritation from sordes in the primæ viæ, as well as from the disordered action of the liver, it will be proper to give a larger dose than usual, following it up in two or three hours with a brisk cathartic. But the active purging thus induced, Dr. A. thinks, should not be repeated by means of calomel; other and more common purgatives being proper.

"In general, it will be found that active purging is rarely required to be repeated in the severest form of the disorder, the principal object in the treatment being to restore the healthful function of the liver, and to procure, once or twice daily, a regular and free, but not a purgative evacuation from the bowels."

It is neither necessary nor proper to allow the medicine to affect the mouth. When it has been employed for some time, and the patient is verging towards convalescence, yet the symptoms of biliary derangement become stationary, or even return, as evinced by the unnatural appearance of the motions, then mercurial friction on the hepatic region employed every night will answer better than an obstinate perseverance in the calomel, the same precautions being attended to in guarding the system from its salivary effects. It is hardly necessary to observe, that the stools should be accurately and daily examined by the practitioner, in order to ascertain the changes which are produced upon the function of the liver by the medicine.

It has been observed, that purgatives are seldom necessary in marasmus, the only purpose, in ordinary cases, that is required from them, being that of merely removing irritating matters from the bowels. Where they are required, therefore, our author chiefly employs the different purgative salts, which he prefers on account of their action being principally exerted on the small intestines, and from their having the property of increasing the secretions of the bowels. He gives them in small doses largely diluted with water. Generally speaking, however, the saline purgatives prove too inert in children, and require to be alternated or combined with calomel. Bitters and tonics, our author wisely condemns. They are injurious in the early stages of the disease, and unnecessary in convalescence. In the few cases where they may be necessary, they are advantageously combined with diuretics. Emetics are only useful at an early period, and then merely to clear the stomach from irritating matters. A free hæmorrhoïdal discharge, occuring in this disorder, has proved serviceable, according to our author's experience; but where he could have seen a communication in a Medical Journal, "in which a practice of introducing leeches *into* the rectum, is noticed as being pursued by the continental practitioners," we are at loss to conceive. Probably Dr. Ayre alludes to the paper of Dr. Brown, in a late number of the Edinburgh Journal; but there is nothing there stated of such a practice as introducing leeches into the rectum. Indeed, we know a serious accident that has resulted from a couple of leeches making a forced entry into the channel in question; and we all know the serious consequences which were felt by the French soldiery in Syria, by the lodgment of leeches in the fauces. The continental physicians order leeches *to* the anus, but not *into* the rectum.

We agree with Dr. Ayre that general and local bleeding in marasmus is of little service, unless where the head is threatened. The great and paramount object is to restore the function of the liver. Opiates are condemned by Dr. Ayre, as also those various remedies which are exhibited for the purpose of allaying the cough, since this *symptom* will only subside with the cause on which it depends. The spontaneous diarrhœa which occasionally arises in this complaint is not to be treated with opiates and astringents, which are sure to aggravate the disease.

"By restoring the secretion of bile, which corrects this putrid and acid tendency in the ingesta, and aiding the descent of its fæculent parts through the bowels, the morbid stimulus which produced the diarrhœa, is no longer given, and the diarrhœa itself is removed." P. 178.

The febrile symptoms are removed by the same means which correct the morbid action of the liver.

"A small dose of calomel, in fact, taken at bed-time, having more effect in subduing the fever and procuring rest than any combination of those medicines which are in the highest estimation for those purposes." P. 180.

The aphthous affections of the mouth are removable by the calomel, when they are dependent on biliary derangements. In respect to food, there should be laid down a precise code of instructions. In marasmus, children generally dislike their accustomed meal of bread and milk in a morning; but on the accession of the febrile stage, they often demand cold milk with avidity, for the purpose of allaying the thirst, by which they considerably increase the disorder.

"In every stage of this complaint, the milk of cows appears to act unfavourably."

The plainest and simplest food is here, as elsewhere, the most proper. The principle on which a cautious and somewhat abstemious regimen is recommended, is founded on the imperfect powers of the stomach, and not upon any *organic* disease of the liver, which is erroneously believed to exist as the cause of the disorder.

"When the stomach becomes equal to the digestion of a full and nourishing diet, with a moderate allowance of wine, there is a positive advantage in allowing it; since it seems to strengthen, in a very material degree, both the stomach and the system, thereby more readily confirming the recovery of the patient." P. 185.

Of the propriety of the above rule, we have much doubt, and not without some little pretensions to experience. Let it be remembered that functional disorder weakens the structure of an organ; and that there is not seldom danger in pouring a rich tide of nutriment through the vascular system of such parts, for a considerable time after the functional disorder has disappeared.

Here Dr. Ayre combats the idea of mothers being incapable of nursing their own children.

"On the contrary, there is a feeling of the highest health; the appetite and digestion being vigorous, whilst the supplies of milk are both regular and abundant." P. 187.

Among the auxiliary remedial measures, there is none on which our author so much depends as the shower-bath;

"Or, what is equally useful, and more generally acceptable, the practice of sponging the body with cold water upon first rising in the morning. The power which this possesses of strengthening the system, and of improving the digestion, and of imparting a feeling of health and vigour to a convalescent in this disorder, is often remarkable; the invalid frequently being enabled, under its use, to digest food, which on all former occasions had disagreed with him, and to resist the influence of many of those causes which had before either produced or aggravated his complaint." P. 188.

Vinegar or salt may be added to the water with advantage.

"The clothing of patients in this complaint should be warm, especially about the feet and legs, which are peculiarly liable to become cold, even when other parts of the body perhaps are preternaturally hot." P. 190.

The remainder of the volume, about 60 pages, is taken up with cases illustrative of the text, of which we have given a very full analysis. Although we shall not enter upon these cases, we deem it necessary to say a few words more respecting Cholera Morbus, the received pathology of which is now suffering attacks from several quarters.

"In the first stage [of Cholera] says our author, there is present a congestive state of the liver." "In this stage there is, in fact, a manifest struggle between the oppressive influence of the complaint, and the vital energies of the system; *the copious secretion of bile which is produced being the consequence of those energies acting to excess, to repel and remove it.*" P. 195. "When that secretion of bile is induced which forms the second stage, it is usually in excess, and of an acrid and morbid nature, becoming, when very considerable, the source of other disorders."

The first stage, or that of congestion, is justly considered by our author to be the most dangerous, and to require the most prompt remedial measures. But these measures, Dr. Ayre thinks, should not be wine, cordials, and opiates, as we generally find them.

"In the *first* stage it is the secretion of the bile, which is to be renewed and rendered healthy; and it is only in the *second* or that in which the secretion is excessive, and when, by the excess, the powers of the system have become exhausted, that wine and cordials are admissible or necessary."

Our readers are perfectly aware that Dr. Ayre has already stated that calomel has the power of checking inordinate and augmenting deficient secretion in the liver.

"In the following cases it will be seen that the practice pursued in the treatment of the Cholera Morbus, consists in giving a third or a fourth part of a grain of calomel, according to the age or other circumstances of the patient, every half hour, for five or six successive hours, or until the sickness abates."

Dr. Ayre states, from much experience, that it is seldom necessary to continue the medicine beyond the sixth or eighth dose, excepting in cases of unusual severity. In this place Dr. Ayre inserts the following note.

"Within these few years, a view of the pathology of Cholera Morbus, similar to the above, has been given in an able work on the diseases of tropical climates, by James Johnson, Esq. but which I feel it necessary to observe, I did not see, until directed to it by a marginal note in a work lately published."

The coincidence then of opinion, between three authors, Drs. Armstrong, Ayre and Johnson, gives strong support to the pathology in question.

But however we may be inclined to agree with Dr. Ayre in doctrine on this point, we are not inclined to adopt his practice; and for this reason, that by employing calomel in larger doses, and combined with opium, we check this exhausting disease in one fourth the time that Dr. Ayre, according to his own statement, appears to require.

But our limits are closed. We have delineated Dr. Ayre's work with much pains and care: every reader, therefore, must see the great value and merit of our author's publication. The only fault is diffuseness of style By terse and chosen language Dr. Ayre might have greatly lessened the size or increased the matter of the work; but every author has not such a command of language. We predict, however, that the work will be widely circulated and favourably received in the medical world.

### Surgery.

### VIII.

*Observations on the Symptoms and specific Distinctions of Venereal Diseases; interspersed with Hints for the more effectual Prosecution of the present Inquiry into the Uses and Abuses of Mercury in their Treatment.* By RICHARD CARMICHAEL, M. R. I. A. &c. One vol. 8vo. pp. 221. 1 plate. London, 1818.

A MORE interesting question than the present has not divided the public sentiments for many years—perhaps for some centuries. It behoves all parties then to conduct their investigations with caution, to state the results with candour, and to listen to the facts and opinions of each other without scepticism or ill humour. Over the origin of syphilis, as of the human race itself, a cloud of mystery still hangs. It came among us, nobody knows whence or how; and why should it not make its departure without any ostensible cause? We acknowledged in the first number and first article of this Journal, that, in whatever way it might be accounted for, the Hunterian forms of primary sores were now *comparatively* rare; and, during the last five months, our observations and enquiries in the metropolis, throughout a very wide circle of professional acquaintance, have tended to confirm the remark. There may be some foundation therefore for Mr. Carmichael's idea, "that there are prevailing forms of venereal affections, like prevailing epidemics, at different times, and in different countries—a circumstance that may possibly depend on the importation of fresh infections."

Thus he states that a few years back, and particularly in 1812 and 13, that malignant attendant of the phagedenic disease, the sloughing ulcer, was extremely prevalent in Dublin, but he has not seen it in a single case, either in private or public practice, during the last two years. The medical officers of our fleets can testify how scurvy, malignant contagious ulcers, and pneumonia have alternately visited, with terrible devastation, our wooden walls. We must not therefore condemn our predecessors as having uselessly exhibited mercury in syphilis when it might have been avoided; nor yet, on the other hand, shut our eyes to the accumulating mass of evidence which flows from all quarters respecting the success of the non-mercurial treatment. Time will settle all these points, and till then, credulity and scepticism ought to be kept out of the investigation.

Mr. Carmichael, in the beginning of his work is unnecessarily severe on those who have borrowed the term *syphiloid* to express diseases which resembled syphilis; but the severity is completely turned against himself in the Appendix, where he is forced "to relinquish his opinion that true syphilis differs from other venereal complaints, by always requiring mercury for its cure;" for if this be the case, where is the impropriety of applying *syphiloid* to those forms of the disease which experience has shown to require no mercury, while we retain *syphilitic* for those forms which, though curable, as it is supposed, without the above-mentioned mineral, are yet quickened in their progress to recovery by a judicious use of the same.

Mr. C. states that "a chief object of this publication is to excite the attention of those engaged in the investigation of the question—Whether venereal complaints may be cured without the exhibition of mercury?" and informs us, that since the appearance of recent papers in the affirmative, such has been the dearth of Hunterian chancres in Ireland, that only three well-marked cases have occurred, either in his public or private practice. These three cases were treated without mercury; and we think it judicious to give some account of them in detail.

The first was a private patient. The induration was such as satisfied Mr. C. of the nature of the disease, "*although the surface had more an excoriated than an ulcerated appearance.*" This gentleman had heard of the non-mercurial doctrines, and urged their adoption in the present instance, which was complied with by our author.

"The chancre remained a month stationary, neither increasing nor diminishing; but *at* the expiration of this time a deep excavated ulcer *had* formed on the induration. I therefore did not feel it warrantable to allow him to remain any longer without mercury. That medicine was accordingly exhibited in the form of frictions, which soon induced the ulcer to heal; but upwards of two months elapsed before the callosity was removed." P. 16.

It is not solely for the purpose of pointing out the incongruity which our author has fallen into, in affirming, that a chancre (which, at first, was rather an *excoriation* than an ulcer) continued a month without either "increasing or diminishing," and yet, *in that very period,* had become a "deep excavated ulcer," that we mark in Italics this strange passage; but to remind Mr. Carmichael, that an author who prides himself on accuracy of language, and is pretty severe on others, should not have been guilty of a contradiction and inaccuracy himself. The chancre, in fact, was not stationary; for although it may not have spread, *it deepened*, which is worse, till mercury was used, when a change was evidently produced for the better. No case, therefore, can more strongly support the doctrine we hold, that, even if true chancres disappear *without* mercury, they disappear *quicker* under its use. The ultimate security of the constitution is a matter of future experience; but we would just hint our suspicions that the low regimen and antiphlogistic measures, prescribed in the non-mercurial treatment, may be a principal cause, not only of the disappearance of *primary*, but of the slow developement of *secondary* symptoms. We are not therefore to be too sanguine till a much longer period has elapsed than is yet allowed for the proof of constitutional security.

*Case* 2. "James Murray was admitted May 20, 1818, with an indurated chancre, situated at the junction of the prepuce with the corona glandis. The surface was covered with a small *crust*, but the callosity was of that remarkable kind that terminates abruptly, and feels like a piece of hard cartilage under the skin. He had also the psoriasis syphilitica in distinct patches, each about the size of a sixpence, scattered over his back, breast, forehead, arms, &c.

"According to his statement, the ulcer appeared three months previous to his admission; *but had in a great measure healed in a month after its appearance, without the use of medicine*, leaving however, behind it the callosity, which was constantly covered with the small crust already mentioned. He added, that six weeks afterwards, the eruption began to appear, and that he had taken nine mercurial pills, which did not affect his mouth.

"From the time of his admission till the 15th of June, a period of twenty-six days, no medicine whatever was given to him; during which the callous substance increased in size, and the eruption became more extended by the addition of new spots and the enlargement of those that had first formed. Finding that the disease was not yielding to the powers of the constitution, I determined to make a trial of the sarsaparilla, the decoction of which was ordered for him, with a drachm of the powder thrice a day. Shortly after the exhibition of this medicine, the callosity began to diminish, and the eruption to decline; and so early as the 5th of July, it was noted that the callosity was considerably lessened, and the eruption was of a paler colour. In all respects the patient gradually amended, and at the time this sheet was called for, the callosity was entirely removed, and the eruption had so far diminished as to leave little doubt of its total dispersion." P. 214.

We are inclined to believe that John Hunter would not have allowed that to be a true chancre which "had in a great measure healed in a month without the use of medicine." It certainly was a case in which we should have hesitated to prescribe mercury in any other form than as an alterative.

*Case* 3. Edward O'Brien, admitted the 24th of May, 1818, with a large ulcer extending along the dorsum of the penis, "*from the prepuce almost to the pubis,*" of a livid colour. There was an eruption of spots on the abdomen and epigastric region, exhibiting the characters of psoriasis syphilitica. No mention whatever is made of the previous history or treatment! Quietude, and a poultice to the penis, were the only remedies directed.—June 1. The ulcer extended.—8th. He began to complain of pains in his shoulders, arms, knees, and tibiæ.

"The pain in the latter afterwards became extremely acute, and he could not bear the slightest pressure on those bones. He lost his rest, and became emaciated.—On the 16th of June, the pain and tenderness of the tibia were so very acute, as were also those in his joints, and particularly in his hands and wrists, and the eruption had so far extended, that I conceived that mercury was absolutely necessary for this individual, and I was disposed to order it on the instant: and in this opinion a most attentive and intelligent class of pupils, who watched this as well as all the other venereal cases with the greatest assiduity, seemed anxiously to concur. I am now, however, well pleased that I postponed this intention, and determined upon giving a trial, in the first instance, to sarsaparilla; although I confess, at the time, I had little or no hopes of succeeding by its means. I directed it in the form of powder and decoction in full doses.

"On the 28th, however, to my surprise, a very remarkable amendment was apparent in all his complaints. The ulcer had looked healthy in the middle, and had ceased to extend. The pains and tenderness of the tibiæ were diminished, and the eruption was obviously on the wane. From this period his amendment was regular and decided." P. 217.

The early part of this case's history is so very imperfect, and the subsequent symptoms of such a doubtful character, that we are very far from being convinced that it was truly syphilitic. These three cases, however, in conjunction with the statements lately made by Messrs. Rose, Hennen, and others, have unhinged our author's creed, and rendered a great part of the beginning of the book a memorable instance of the instability of even MEDICAL FACTS! The very same types which advocated the distinction between syphilis and other *venereal* affections, in signature A, were doomed, when they arrived at 2 D, to revoke those sentiments which were erected on "a foundation, not of theory or speculation, caprice or prejudice, but in the honest simplicity of stubborn facts." Yet, while we cannot help thinking that Mr. Carmichael has been somewhat too precipitate "in thus relinquishing his opinion that true syphilis differs from other venereal complaints by always requiring mercury for its cure," we reverence his candour, and highly applaud his zeal. But to return to other practical points in the Essay before us. The second chapter is on Iritis. In our author's former publication, he thought he had "sufficient grounds to attribute venereal inflammation of the iris to the poison of syphilis;" but, since that time, cases have occurred to him, both in public and private practice, which have induced him to change his mind on this subject too. We really think these sudden changes indicate one of two things—*precipitation* in publishing an opinion, or *vacillation* afterwards. They are both very dangerous to an author's fame; and although, in Mr. Carmichael's book, they are only maculæ on a large disk of talent and of merit, yet the very splendour that surrounds them renders them more conspicuous. If we were not convinced that he has too much good sense to be offended at these remarks, we should not have made them.

The cases, which occasioned this change of mind in our author, were attended by a cutaneous eruption, where the spots were papular. He dissents from Mr. Travers relative to *mercurial* iritis; and at page 90 of this volume, will be found a similar dissent on our own parts. In regard to the treatment, Mr. Carmichael confirms the observations of Dr. Farre and Mr. Travers.

"But inflammation of the iris, whether it originates from venereal virus or from any other cause, will readily yield to mercury and the antiphlogistic means. Thus by means of the mercurial action the inflammation is arrested, and the deposition of coagulable lymph is prevented. If, however, it has already taken place, instead of becoming organized, which would render the iris immovable, it is absorbed, through the influence of this medicine, and the other stages of the adhesive inflammation are also prevented."

Mr. C. quotes the authority of Dr. Thomson of Edinburgh, in proof that mercury is not *absolutely* necessary for the removal of iritis, as the latter gentleman cured seven cases without its exhibition. Our author next details eleven cases of iritis, coming in the end to the following conclusions.

"From all the preceding cases, we learn many important facts, and in the first place, that iritis is an attendant on the papular eruption. By cases one and five, it is ascertained that the papular eruption will occur either after alterative or full courses of mercury. By cases 1, 8, 9, 10, and 11, that gonorrhœa alone is sufficient to produce the papular eruption. By cases 7, 8, 9, 10, 11, that iritis will occur where little mercury, or none at all has been employed, and therefore it cannot be attributed to that medicine. All the cases prove the utility of combining the depleting with the mercurial plan for the cure of iritis. But the mercury was, in every instance, discontinued as soon as the mouth became affected."

The 3d Chapter is "on the primary ulcer, with elevated edges, and its constitutional symptoms." This ulcer is of a chronic nature, and borders closely on the phagedenic ulcer, described by Mr. Carmichael in a former work. The distinction is of no *practical* consequence, as the treatment is the same. We shall pass this chapter over, as it consists almost entirely of a re-publication of the cases inserted in the Medical and Physical Journal about two years ago.

### Chap. IV. *On the Primary Phagedenic Ulcer.*

This constitutes a disease of very uncertain prognosis—tedious under the most judicious treatment—destructive to the constitution, or even life of the patient, if treated with mercury. The sloughing or phagedenic ulcer is described by Celsus, while the chancre was not known in Europe until the 15th century. In the *former*, the eruption is formed of pustules or hard tubercles, which rapidly scab or ulcerate. In the chancrous form, it is scaly from the commencement, and does not ulcerate until after several weeks or months have elapsed. Mercury in the *first* is, according to our author, injurious, or at least uncertain in its effects; in the *second*, it is always beneficial. After the healing of the phagedenic ulcer, however, Mr. C. does not object to a moderate use of mercury, for the purpose of guarding against constitutional attacks.

### Chap. V. *Constitutional Symptoms of the Phagedenic Ulcer.*

The eruption in these cases is frequently ushered in by a high degree of fever, followed by tubercles or spots of a pustular tendency; both of which quickly degenerate into ulcers covered with thick crusts, which heal in a peculiar manner from the centre, while at the same time they are extending at their circumference with a phagedenic border.

"All eruptive diseases, accompanied by fever, are attended by affection of the throat; but it cannot be expected that these affections will afford as decisive a diagnosis as the eruptions. This remark particularly applies to venereal diseases: the affections of the throat in them bear so far a resemblance to each other, that it would be presumption to decide on the nature of the disease by this symptom alone, but when combined with others, it will considerably assist our judgment." 78.

Thus, if with a deep excavation of the tonsil, there be a *scaly* eruption which is so characteristic of Syphilis, we need not hesitate about the nature of the complaint or the exhibition of mercury. On the other hand, if the patient complains of soreness and rawness in the fauces, accompanied by chronic swelling of the tonsils; and moreover if, upon examination, an eruption of *papulæ* be discovered, with pains in the larger joints, then these secondary symptoms are to be looked upon as resulting from what our author denominates *venereal* but not *syphilitic* poison, and mercury is to be avoided.

The affection of the throat following phagedenic primary ulcer, is of the most formidable and destructive nature. It spares no part of the fauces, but the back of the pharynx is its principal theatre. The ulcer is usually covered with a thick, white, tenaceous, slimy matter, and it is attended with a considerable difficulty in deglutition. It requires the most prompt means to arrest its progress; for if it reach the larynx, there is little hope of saving the patient. This ulcer frequently extends upwards into the nares, occasioning fœtid breath and a foul discharge from the nostrils, followed by exfoliations of the bones and sinking in of the cartilages. Mr. C. never witnessed an instance of this kind conjoined with the papular eruption, or the *scaly syphilitic lepra,* or psoriasis. We have; and many of those who read this Journal will recognize the distressing case we particularly allude to.

Mr. C. thinks that this train of symptoms is so very often found to be injured, rather than benefited, by the exhibition of mercury, "that we may ascribe the coinage of the favourite terms mercurial, (in the unrestricted sense in which it is employed) syphiloidal, sequelæ, &c." P. 82.

Our author has no doubt but the embarrassing obstinacy of this disease perpetually arises from the premature and indiscreet interposal of mercury, interrupting its natural progress.

"I may therefore be permitted to argue, that the mercury which interrupts the natural progress of the morbid poison, and often removes the eruption, and heals the constitutional ulcers of the skin and throat, transfers the disease to the deep seated-parts—the periosteum, fascia, and bones." P. 85.

This inference, he thinks, is supported by the fact that the bones are seldom or never affected in those who have not used mercury. He compares the disease under consideration to yaws, both as to nature, treatment, and deterioration by mercury. Mr. C. acknowledges, however, that mercury may be had recourse to under the following circumstances. 1st. When the ulcer is making such rapid strides as to endanger the parts of the throat on which it is working. Here it is better to hazard the consequences which ensue from interrupting the natural progress of the disease, than to allow the destruction of parts essential to the comforts or life of the patient.

2d. When the disease has existed a long time, and, although lingering, is evidently yielding to the powers of the constitution. Here mercury will not interrupt the operations of Nature, but rather accelerate their salutary career.

3d. If nodes and other affections of the deep-seated parts should not appear to yield to decoction of the woods, and other means, we must then have recourse to mercurial assistance.

The constitutional treatment recommended by Mr. Carmichael for those symptoms which follow phagedenic ulcer are, the strong decoctions, simple or compound, of sarsaparilla, conjoined with the extract or powder of the same plant, or with antimonials, or the compound powder of ipecacuan, in such doses as the stomach will bear. Mr. C. speaks favourably of the decoction of guaiacum wood, in conjunction with pills of gum guaiacum and antimonial powder. These medicines, as they increase the secretions, so they require confinement on the part of the patient. Local inflammation and swelling, especially about the joints, require, of course, those local detractions of blood and other means which experience has proved to be useful.

"If the patient is affected with extensive irritable constitutional ulcers, with phagedenic edges, great advantge may be derived from combining with the decoction of the woods the exhibition of cicuta either in powder or extract, in full doses. If he is covered with numerous scabby spots, and interspersed with trifling superficial sores, which are the secondary appearances that follow the primary *ulcer with elevated edges*, the daily use of the bath, impregnated with sulphurated kali, will be found extremely useful."

Where great irritability prevails, the bread and water poultice, or the black wash (calomel and lime water) will be useful. For the large tubercles, Mr. C. recommends, in strong terms, the nitrous acid internally. To the ulcers in the throat, Mr. C. applies (and we can confirm his recommendation) stimulating applications, as the nitrate of ilver; six or ten grains to the ounce of water, or the muriate of mercury three or four grains to the ounce of water. Should the ulcers still spread, then mercury, as a last resource, is to be used to stop their progress. The preparations Mr. C. employs are the ointment and fumigations of factitious cinnabar to the throat, applied three or four times a day.

"I can safely aver that I never met with any ulcer of the throat, however extensive and malignant, that has not yielded to one or other of these methods, if the larynx was free from disease."

To chronic nodes he repeatedly applies blisters with the internal use of the woods.

If the disease be evidently on the decline, but chronic and lingering, then a mercurial course may be adviseable. When the skin or the throat is affected, Mr. C. usually prescribes the oxymur. hyd. or the calomel and antimony pills, in conjunction with the decoct. sarsæ. Where the patient is delicate, the decoction and blue pill; where there are nodes or other affections of deep seated parts, a full mercurial course is necessary for the purpose of influencing their low organization; and that is best done by mercurial frictions. Our author here selects "from many volumes of notes," a sufficient assemblage of cases to satisfy any reasonable mind of the decided advantage to be found in the treatment here recommended. To these cases we refer, as our limits prevent our extracting any.

The 7th Chapter is on "Anomalous Disorders, resembling the Phagedenic Disease, yet not of Venereal Origin." Our author, since his first publication, has met with a great variety of these instances. He dissents in some measure from Mr. Abernethy's plan of attempting their cure, especially when they are spreading rapidly in the throat, for instance, by attention only to the digestive organs. He would arrest their progress, as in the other cases, by mercury carried to saturation of the system; though, under ordinary circumstances, small doses of the blue pill with sarsaparilla are sufficient.

Painful nodes or affections of the periosteum, in persons who declare they never had any venereal disease, are so frequently met with, that Mr. Carmichael has not thought it necessary to note any cases in elucidation. The general treatment which he has found most successful is the exhibition of those remedies already enumerated, while the local treatment consists in the application of leeches, followed by blisters to the part; and if these fail, a division of the periosteum. We shall here, notwithstanding our closing columns, introduce an instance which proves the close resemblance between venereal and non-venereal affections.

"On the 12th of June, 1815, I was called upon to see a young unmarried lady, whose condition and morals placed her altogether beyond the reach of suspicion; yet the symptoms with which she was affected precisely resembled those which are undoubtedly of venereal origin. There was a considerable number of large tubercles, similar to those described in the last chapter, scattered over her legs, arms, and thighs, attended with discolouration of the integuments. She complained of soreness of her throat; and, on examination, I found the back of the pharynx ulcerated and covered with white tenacious matter, evincing a similar correspondence between the affection of the skin and throat to that which I had often witnessed in venereal cases. Her tongue was white and furred, with bad appetite, and general derangement of the system. I merely regulated her diet, ordered her five grains of the blue pill every night, and three drachms of the sulph. magnes. every morning, under which plan she perfectly recovered before the 12th of July following."

The 8th, or concluding Chapter, is a warm remonstrance with Mr. Charles Bell, in which our author advocates with great zeal, the talents and respectability of our army brethren—a cause, indeed, which requires little support: for if any man ask where are the proofs of those talents, he may be answered—*Circumspice!* The period is arrived when the medical officers of our armies and of our fleets shall teach their brethren in private life to *respect* them, as their companions in arms have taught their enemies to *fear* them. They have shared the toils, and the dangers, and the privations of war, with the sole reward of laurels and experience; they have now, though some of them at the *eleventh hour*, entered the vineyard of peace, and, notwithstanding the ungenerous envy of some of their fellow-labourers, they will be rewarded by a liberal master—the Public.

The reader will, ere this, have formed his judgment of the value of this volume. Mr. Carmichael's reputation as an author and able practitioner is established; but we would just hint at the unnecessary extent to which the present work has been pushed by multiplication of *cases*. Such, however, is the prevailing taste of the day. But with this exception, the work is extremely valuable; and as such we can confidently recommend it to our surgical brethren.

### CATALOGUE RAISONNÉE.

### I.

*Practical Illustrations of Scarlet Fever, Measles, and Pulmonary Consumption; with Observations on the Efficacy of Sulphureous Waters in Chronic Complaints.* By JOHN ARMSTRONG, M. D. Physician to the Fever Institution of London. *Second Edition.*

Two large impressions of a medical work in the same year, afford pretty unequivocal proof of the state of professional feeling towards its general merits. We have only room to say, that considerable improvements are introduced into the Second Edition, particularly in the Section on *Chronic Diseases,* which now forms a prominent feature of the work. To recommend the Publication, after what we have said in the first quarterly number of this Journal, would be superfluous.

### II.

*A Supplement to the Pharmacopœias; including not only the Drugs and Compounds which are used by Professional and Private Practitioners of Medicine; but also those which are sold by Chemists, Druggists, and Herbalists, for other Purposes; together with a Collection of the most usual Medical Formulæ; an Explanation of the Contractions used by Physicians and Druggists; the Medical Arrangement of the Articles of the London Pharmacopœia, with their Doses at one View; a similar List of the Indigenous Plants of the British Islands, which are capable of being used in Medicine: and also a very copious Index, English and Latin, of the various Names by which the Articles have been known at different Periods.* By SAMUEL FREDERICK GRAY, Lecturer on the Materia Medica, &c. London, pp. 390.

THIS is a desperate long title; and on reading it over, we anticipated a Spanish Bill of Fare, but were agreeably disappointed. Mr. Gray has actually performed what his title page indicates, and a great deal more. The labour bestowed on this compilation must have been immense, and the only faults we have to find are, first, a not very happy arrangement; and secondly, a bluntness in his pruning hook, by which accident a great number of useless shrubs, plants, or rather *weeds,* are permitted to intermingle with those which deserve cultivation and manure. Still there is great merit in the work; and as the title page delineates the prominent features of the book very accurately, while it is one that cannot be analysed, we have given it entire, recommending it to the libraries of those to whom it is addressed, as a Companion or a Glossary, rather than "a Supplement to the Pharmacopœias."

### III.

*A Letter from a Physician in the Highlands to his Friend in London, on the Subject of a Consumptive Habit.*

There are many judicious observations in this little Pamphlet; all hinging, however, on temperance, in its most extended sense, as the grand prophylactic of Pulmonary Consumption.

### IV.

*A Manual of Practical Anatomy for the Use of Students engaged in Dissections.* By Edward Stanley, Assistant-Surgeon and Demonstrator of Anatomy at Bartholomew's Hospital. One Vol. Octavo, pp. 439. 1818.

Mr. Stanley is humble in his claims; yet, his labours may not be the less meritorious. He arrogates no other praise than that of arranging the several regions of the body in the order most convenient for their examination, and of describing each part successively, according to its natural situation. He has left out the *uses* of the muscles, as being a branch of physiology, by which means he has been enabled to give a greater development to their anatomy. This plan we think judicious. We have dipped into several anatomical descriptions in Mr. Stanley's work, and find them clear, sufficiently copious, yet explicit.

### V.

*Anatomical Examinations. A Series of Questions, with Answers, &c. &c. intended as preparatory to Examination at the Royal College of Surgeons, &c.* Two Vols. Octavo, 4th Edition. 1818.

We are not very friendly to grinding and cramming; and consequently we view with a jealous eye every one of those near cuts to the Temple of Science, denominated Vade-Mecums, Examinations, &c. But as we have generally observed full two-thirds of our fellow travellers *scampering* along these lanes and by-ways, in hopes of getting before the plodding book-worms, who take the long and circuitous route to the summit of the hill, so we think it lost time to attempt to persuade them that they are in the wrong track. To all those then, who are doomed to pass under the yoke of Machaon and Podalirius in Lincoln's-Inn Fields, we recommend a month's previous study of these volumes, as a complete *anti-micturant* in the portico of that dreaded temple; and, as an Aladdin's lamp, that will light them safely past every Cerberus, Cyclop, and Medusa, which they may encounter within those gloomy walls.

### VI.

*Strictures on Parish Registers and the Bills of Mortality, &c.* By G. M. BURROWS, M. D. &c. &c. &c. Octavo, sewed, London, 1818.

THIS Pamphlet, which is equally interesting to the Philosopher, the Physician, and the Politician, has impressed us with a high sense of Dr. Burrows' powers, as a close and deep reasoner on every subject to which he directs his attention. It will well repay the perusal.

### VII.

*Memoirs of the Life and Doctrines of the late John Hunter, Esq.* By JOSEPH ADAMS, M. D. &c. &c. Second Edition, corrected by the Author. One Vol. 8vo. pp. 262, with an Engraving. London, 1818.

SUCH was the modesty or the diffidence of the late lamented Dr. Joseph Adams, that the small edition which he published of the above work, did not nearly satisfy the first subscription of the trade. In consequence of this, another Edition was put to press; and just as the work was finished, a premature death snatched the Editor from the scene of his useful and honourable labours!

To a liberal British public we have only to say, that the present Edition, corrected and enlarged, is at the risk of the Widow of him whom the Profession esteemed.

### VIII.

*Introduction to a Course of Lectures on the Diseases and Operative Surgery of the Eye.* By WILLIAM M'KENZIE, Member of the Royal College of Surgeons. pp. 34, sewed. London, 1818.

MR. M'KENZIE is a very intelligent and well informed young man, who has travelled and studied much on the Continent; and in directing his attention to the pathology and operations of the eye, has qualified himself remarkably well for instructing the rising generation of pupils in a very important branch of their profession. The Introduction is characterized by great modesty and perspicuity; and we understand from his pupils, that his Lectures on the Eye are extremely interesting, and remarkably well adapted to convey to, and impress on the young mind, a thorough knowledge of the subject.

